# Support Vector Machine Classification of Major Depressive Disorder Using Diffusion-Weighted Neuroimaging and Graph Theory

**DOI:** 10.3389/fpsyt.2015.00021

**Published:** 2015-02-18

**Authors:** Matthew D. Sacchet, Gautam Prasad, Lara C. Foland-Ross, Paul M. Thompson, Ian H. Gotlib

**Affiliations:** ^1^Neurosciences Program, Stanford University, Stanford, CA, USA; ^2^Department of Psychology, Stanford University, Stanford, CA, USA; ^3^Imaging Genetics Center, Institute for Neuroimaging and Informatics, Keck School of Medicine of the University of Southern California, Los Angeles, CA, USA

**Keywords:** major depressive disorder, diffusion-weighted imaging, graph theory, support vector machine, small world network, subgenual anterior cingulate cortex

## Abstract

Recently, there has been considerable interest in understanding brain networks in major depressive disorder (MDD). Neural pathways can be tracked in the living brain using diffusion-weighted imaging (DWI); graph theory can then be used to study properties of the resulting fiber networks. To date, global abnormalities have not been reported in tractography-based graph metrics in MDD, so we used a machine learning approach based on “support vector machines” to differentiate depressed from healthy individuals based on multiple brain network properties. We also assessed how important specific graph metrics were for this differentiation. Finally, we conducted a local graph analysis to identify abnormal connectivity at specific nodes of the network. We were able to classify depression using whole-brain graph metrics. Small-worldness was the most useful graph metric for classification. The right *pars orbitalis*, right inferior parietal cortex, and left rostral anterior cingulate all showed abnormal network connectivity in MDD. This is the first use of structural global graph metrics to classify depressed individuals. These findings highlight the importance of future research to understand network properties in depression across imaging modalities, improve classification results, and relate network alterations to psychiatric symptoms, medication, and comorbidities.

## Introduction

Major depressive disorder (MDD) is among the most common psychiatric disorders in the world, affecting more than 350 million individuals (1), and is associated with a large and increasing economic and personal burden (2). MDD is characterized by low mood and loss of pleasure (anhedonia); other significant symptoms involve difficulties in motivation, attention, psychomotor functioning, sleep, and appetite. With the advent of tools and procedures to assess human brains *in vivo*, the neuroscience of MDD has experienced tremendous growth over the past two decades. While early work in this area documented anomalies in specific structures in MDD (3, 4), more recently investigators have begun to examine brain networks (5–8). Initial evidence from this literature indicates that MDD is associated with abnormalities in both structural and functional networks [for reviews, see Ref. (5–8)]. More specifically, MDD is associated with abnormal resting-state functional connectivity in a cortico-limbic (prefrontal–amygdala–pallidostriatal–mediothalamic) mood-regulating circuit and in the default-mode network [DMN; (5)]. MDD is also characterized by structural abnormalities in white matter regions that link prefrontal cognitive control areas with subcortical emotion processing regions (8).

In this context, diffusion-weighted imaging (DWI) can be used to assess water diffusion in the brain and is the most widely used tool for assessing white matter connectivity in MDD. Using image analysis methods, this diffusion information can be used to track neural pathways in 3D models. The most commonly used model of diffusion is diffusion tensor imaging (DTI), which uses tensors to quantify the rate of water diffusion for a given voxel in three principal directions. This tensor information can then be used to track neural pathways algorithmically. More sophisticated diffusion models can use high-angular resolution diffusion imaging (HARDI), making it easier to characterize complex white matter anatomy, including crossing fibers. White matter fibers can be grouped into whole-brain networks that can be examined using graph theory (9, 10), which represents network-level properties of the brain. In this approach, researchers create a graph representing the brain using various brain regions as “nodes,” with edges (i.e., connections between them) computed from either correlated activation in resting-state functional magnetic resonance imaging (rs-fMRI), or from properties of fibers computed using tractography in DWI. Characteristics of the resulting graph can then be summarized using continuous metrics to describe large-scale network properties.

Given the interest in how brain regions interact in MDD, several studies have used graph metrics to study such relations. Three studies have used graph metrics to analyze structural connectivity with DWI; none of these studies found global network abnormalities in MDD participants (11–13). Although global graph metrics may not yield MDD-related abnormalities when examined individually, multivariate methods may identify abnormal *patterns* of global graph metrics associated with this disorder. In the present study, we used linear support vector machines [SVMs; (14)] to differentiate MDD participants from healthy controls using structural graph metrics. Using an exhaustive feature scoring technique and feature weight ranking, we also examined which graph metrics contributed most strongly to the differentiation of depressed from non-depressed individuals. We then related the most robust graph metric to clinical measures (i.e., depression severity, level of global functioning, age of onset of depression, and years since onset). Finally, we conducted a regional graph analysis of *degree centrality* (i.e., the level of network connectivity of each given brain region) to understand more precisely how the network connectivity of specific brain regions may be abnormal in MDD.

This study had four aims: (1) use global graph metrics in conjunction with SVM to differentiate depressed from healthy individuals; (2) characterize the ability of specific graph metrics to classify depression; (3) understand the relations between characteristics of the onset and severity of depression and global graph metrics; and (4) examine local network properties that may contribute to global network abnormalities.

## Materials and Methods

### Participants

Thirty-two participants, all women aged 18–55 years, were included in the current study (14 diagnosed with MDD). All participants were recruited using online postings describing participation in a paid research study at a major local university. Psychiatric diagnoses were established using DSM-IV-TR criteria assessed with the Structured Clinical Interview for DSM Axis I [SCID-I; (15)], and the 17-item Hamilton Depression Rating Scale (HAM-D) was administered to assess severity of the depressive episode (16). All participants in the MDD group were currently experiencing a diagnosable depressive episode. Participants in the control (CTL) group did not meet criteria for any past or current Axis I disorder. Exclusion criteria for both the CTL and MDD group included current alcohol/substance abuse or dependence, history of head trauma with loss of consciousness >5 min, aneurysm, or any neurological or metabolic disorders that require ongoing medication or that may affect the central nervous system (including thyroid disease, diabetes, epilepsy or other seizures, or multiple sclerosis). Level of education was quantified using an 8-point scale (from 1 = completed elementary education to 8 = completed professional or graduate education). Depression severity was assessed on the day of MRI data acquisition using the Beck Depression Inventory-II [BDI-II; (17)]. Participants’ age at first onset of depression was assessed during the SCID-I. Years since the first episode of depression was computed as the difference between the participant’s current age and age at onset. Finally, participants were administered the Global Assessment of Functioning [GAF; (18)], a 100-point scale that indexes their level of social, occupational, and psychological functioning. Each participant provided written informed consent, and the study was approved by the Stanford University Institution Review Board.

### Neuroimaging data acquisition

Magnetic resonance imaging data were acquired using a Discovery MR750 3.0 T MR system (GE Medical Systems, Milwaukee, WI, USA) at the Stanford Center for Neurobiological Imaging. Whole-brain T1-weighted images were collected using a sagittal spoiled gradient echo (SPGR) pulse sequence [repetition time (TR) = 6240 ms; echo time (TE) = 2.34 ms; flip angle = 12°; spatial resolution = 0.9 mm × 0.9 mm × 0.9 mm; slice number = 186; scan duration = 315 s]. The T1-weighted images were used for anatomical segmentation and localization. Diffusion-weighted images were acquired using a single-shot, dual-spin-echo, echo-planar imaging sequence [96 unique directions; *b* = 2000 s/mm^2^; TR = 8500; TE = 93.6 ms; spatial resolution = 2 mm × 2 mm × 2 mm; slice number = 64; scan duration = 901 s) and included nine non-diffusion-weighted (*b* = 0 s/mm^2^) volumes.

### MRI data preprocessing

Raw diffusion data were processed using the FMRIB Software Library’s (FSL)^1^ *eddy_correct* tool for eddy and motion correction. Fractional anisotropy (FA) was computed on a voxel-wise basis using a single-tensor diffusion model (19, 20). An optimized global probabilistic tractography method (21, 22) was used to estimate whole-brain tractography. A total of 45,000 fibers were estimated for each participant. FreeSurfer^2^ was used to segment the T1-weighted images according to the Desikan–Killiany method (23). FreeSurfer processing was visually inspected for major errors. No manual edits were conducted (24, 25). This resulted in 68 unique cortical regions per participant (34 per hemisphere; for complete list, see Table 1). Cortical regions were dilated to increase their intersection with white matter, and to make it easier to create tractography-based connectivity matrices. The T1-weighted images were then registered to the FA image (in native diffusion space) using an affine followed by a non-linear transformation via the automatic registration toolkit (ART) (26, 27). The resulting transformations were then used to warp the dilated cortical segmentations to native diffusion space.

**Table 1:** **Complete list of cortical regions of interest (ROIs)**.

	Cortical region
1	Banks of the superior temporal sulcus
2	Caudal anterior cingulate
3	Caudal middle frontal
4	Cuneus
5	Entorhinal
6	Frontal pole
7	Fusiform
8	Inferior parietal
9	Inferior temporal
10	Insula
11	Isthmus of the cingulate
12	Lateral occipital
13	Lateral orbitofrontal
14	Lingual
15	Medial orbitofrontal
16	Middle temporal
17	Parahippocampal
18	Paracentral
19	*Pars opercularis*
20	*Pars orbitalis*
21	*Pars triangularis*
22	Peri-calcarine
23	Postcentral
24	Posterior cingulate
25	Precentral
26	Precuneus
27	Rostral anterior cingulate
28	Rostral middle frontal
29	Superior frontal
30	Superior parietal
31	Superior temporal
32	Supra-marginal
33	Temporal pole
34	Transverse temporal

*Note that each ROI appears in both left and right hemisphere*.

### Creation of connectivity matrices

For each participant, in native diffusion space, connectivity matrices were created using the dilated cortical regions from the Desikan–Killiany atlas as nodes and the number of fibers connecting each pair of regions as edge weights. This resulted in a 68 × 68 connectivity matrix for each participant, with each row and column representing a cortical region, and each cell element representing an edge between the corresponding cortical regions. Edge values were normalized such that the minimum edge value was 0, and the maximum 1. This removed the potential influence of the number of fibers connecting pairs of regions across individuals. To ensure that the same number of connections were present in each participant’s connectivity matrix, we applied a sparsity threshold to the connectivity matrices so that only the 25% most robust edge weights were retained. A value of 25% was used for sparsity thresholding because this value falls within a biologically plausible range (28) and several graph metrics (e.g., *global efficiency*, *characteristic path length*) have been found to be unreliable at sparsity thresholds lower than 25% (29). Although there exist other methods for the selection of sparsity thresholds [e.g., area under the curve (AUC)], it is currently unclear which selection method is optimal (30). Finally, the matrices were binarized such that non-zero remaining edge weights were set to 1. This resulted in a binarized, undirected graph. All graph analyses were conducted using the Brain Connectivity Toolbox [BCT; (31)] in MATLAB (the Mathworks, Natick, MA, USA).

### Whole-brain graph metric computation

Nine graph metrics were selected as features for SVM classification based on their ability to characterize whole-brain network-level characteristics (31–35): *assortativity*, *global flow coefficient*, *global total flow*, *global betweenness*, *global efficiency*, *modularity*, *characteristic path length*, *transitivity*, and *small-worldness*. All nine global graph metrics were computed from the undirected, binarized matrices.

Here, we describe the nine global graph metrics that we used for classification. For further details including equations, see Rubinov and Sporns (31) unless stated otherwise. *Assortativity* is the correlation coefficient between *degrees* of all nodes on opposite ends of a link. High *Assortativity* indicates that vertices of a relative *degree* (connectedness) tend to connect to vertices with similar *degree*. *Global flow coefficient* is the average *flow coefficient* over the network, where *flow coefficient* is defined as the number of all paths of length two linking neighbors of a central node that pass through the node, divided by the total number of all possible such paths (32). *Global total flow* is the average number of paths that *flow* across the networks nodes (32). *Betweenness centrality* of a given node is the fraction of shortest paths in a network that include that node. *Global betweenness* was computed as the average node *betweenness centrality* of the given network [as in Ref. (33)]. High *global betweenness* indicates that nodes of the given network participate in a large number of shortest paths. *Global efficiency* is the average inverse shortest path length in a network and indexes how well a network can transmit information at a global level. *Modularity* quantifies the degree to which a network can be subdivided into clearly delineated sub-networks. *Modularity* offers insight into the community structure of the given graph. Due to variation in the algorithm, *modularity* was computed as the average value across 10 iterations. *Characteristic path length* is the global mean of the distance matrix, which is a matrix of shortest paths between pairs of nodes. Thus, *characteristic path length* is the average shortest path length of the given network, which offers an index summarizing the connectedness of a matrix. *Transitivity* is the ratio of triangles (set of three nodes that each connects to the other two) to triplets (three nodes that are not fully connected) in a network. *Transitivity* measures the degree to which nodes in a graph tend to cluster and is a version of the *clustering coefficient*. We used *Transitivity* instead of *clustering coefficient* as in the computation of *clustering coefficient* the mean *clustering coefficient* is normalized for each node, which may inflate the importance of low degree nodes. Contrastingly, *transitivity* is normalized collectively and therefore is not susceptible to this issue (34). Finally, *small-worldness* was calculated as the ratio of *transitivity* to *characteristic path length* and indexes the balance between local specialization and global integration (35). *Small-worldness* was computed using *transitivity* and *characteristic path length* each normalized against 10 instances of the given metric computed from randomized versions of the original binarized matrices that maintained the degree distribution of the original binarized matrices.

These nine metrics assess important global network properties, including integration, segregation, resilience, and the balance of integration and segregation. Seven of these metrics were selected on the basis of their widespread usage (31), while both *flow*-related metrics were included in order to yield complementary insight concerning integration: *flow coefficient* is similar to *global betweenness*, but utilizes only local-level information (i.e., constrained to the first shell of the given node, and paths of maximum length two).

We used permutation-based, two-sample, two-tailed *t*-tests to compare global graph metrics between the depressed and control groups. Specifically, we computed a *p*-value based on the percentage of *p*-values from 100,000 random shuffles of group labels that were less than or equal to the original *p*-value associated with veridical group labels. To control for false positive inflation as a result of conducting nine statistical tests, we used a false discovery rate (FDR) procedure with *q* = 0.05 (36).

### SVM classification

Support vector machines is a method for supervised classification developed in the field of machine learning that uses training data to learn a classifier (i.e., the parameters of a classification function), which can then be used to classify novel, “test,” data (14). SVM constructs a hyperplane (i.e., high-dimensional plane) to robustly separate groups in *m*-dimensional feature space, where *m* is the number of features. To assess classifier performance, we used leave-one-out cross-validation, averaging performance across *N* folds. Leave-one-out cross-validation is a form of *k*-fold cross-validation where *k* is equal to *N* and is a commonly used method for the classification of psychiatric disorders (37–39). In each fold of the cross-validation, the individuals are grouped into disjoint training and testing sets such that there are no subjects used for both training and testing in a single fold. This process is repeated *N* times and the results from all the folds are averaged to obtain a final estimate of accuracy (40). This cross-validation design was used for every result related to classifier performance presented in this work, and was used to evaluate the generalizability of the classifier given that there is a separate test set in each fold. Although we have a relatively small sample size in this study, we do have the advantage in our analysis of including 3.6 times as many individuals as features; this is a high ratio that should help to reduce the classification error. All SVM-related analyses were conducted in MATLAB.

Specifically, here we used linear SVM, generalized to non-separable training data (14), to classify individuals diagnosed with depression vs. healthy controls, using graph metrics as the features. Explicitly, the optimal hyperplane was defined by:

(1) $$\left\langle w,x\right\rangle +b=0$$

where *x_i_* ∈ ℜ*^d^* represents graph metric feature vectors with length *d*, and *w* ∈ ℜ*^d^* separates the groups (i.e., classes) by maximizing the margin between the hyperplane and each group. The optimal hyperplane is identified using the L2-norm problem:

(2) $$\begin{array}{c} \mathrm{argmin}\\ w,b,v \end{array}\left(\frac{1}{2}\left\langle w,w\right\rangle +D\sum_{i}v_{i}^{2}\right)$$

with the following constraints:

(3) $$\begin{array}{c} y_{i}\left(\left\langle w,x_{i}\right\rangle +b\right)\geq 1-v_{i}\\ v_{i}\geq 0 \end{array}$$

where *D* is a penalty parameter, *v_i_* represent slack variables, and *y* = ±1 represents group label with −1 for depressed, and 1 for control. The value of *D* was scaled for each data point based on group size, that is:

(4) $$D=\frac{N}{\left(2\times N_{G}\right)}$$

Where *N_G_* is the number of individuals in a given data point’s group. Feature weights were computed based on their relation to the hyperplane [i.e., |*w*|; (41)].

### SVM performance evaluation

We assessed in two ways whether graph metrics can be used to classify MDD vs. healthy individuals. First, using the sign test, we assessed the performance of the classifier with all nine global graph metric features. Second, we used a method based on exhaustive feature combination in which we assessed performance across an exhaustive set of classifiers created using combinations of the nine graph metrics. In total, 511 sets of SVMs were trained [i.e., all combinations of the nine features (2^9^ − (null feature set) = 511 unique feature sets)]. For a given set, classification of 22 or more of the 32 folds was considered statistically significant performance (two-tailed sign test, *p* = 0.05). Next, to assess SVM performance across sets, we tested the number of total tests that reached significance against the null hypothesis that would be expected under chance performance; that is, that only 5% of the 511 tests would be expected to reach significance (i.e., two-sided binomial test with alpha = 0.05).

We used two methods in order to yield complementary information regarding classifier performance. The first method relies on information from all nine graph metrics in a single model, and thus offers insight into SVM performance using information across all features. Because feature selection can affect SVM performance, the second method yields information about robustness across features using all possible combinations of features.

### Identifying most robust graph metric

We conducted two analyses to evaluate the robustness of individual graph metrics for classifying depressed vs. healthy individuals. First, we assessed feature weights for each graph metric in the SVM set that included all nine metrics. Feature weights were computed based on the relation of a given feature to the decision boundary (SVM hyperplane), as in De Martino et al. (41). For a given graph metric, the average ranked feature weight was computed across cross-validation folds to assess the relative importance of that metric in classification. Higher ranks indicated greater importance. Ranks were used instead of raw feature weights as raw feature weights have relative units, which may not be consistent across folds. Second, using a technique based on exhaustive feature combination, we aggregated the accuracies that included each graph metric and then compared raw counts reaching significance (i.e., 22 of 32 correct classifications); we then used permutation-based two-sample *t*-tests to test for statistical differences in these accuracies between metrics.

These two methods of feature ranking are complementary. The first method explicitly quantified relations between all features as computed using SVM feature weights (41) from the full model (i.e., the model that included all features). The second incorporated information across all combinations of metrics, focusing on overall performance accuracies associated with a given feature. This approach is helpful for assessing the robustness of a given metric across models while taking into account the influence of feature selection.

### Additional analyses of the most robust graph metric

After identifying the most robust graph metric for classification, we conducted additional analyses in order to better understand this metric in relation to classification and differentiation of the depressed and non-depressed groups, and in relation to clinical variables within the MDD group. Thus, we tested the aggregate accuracies of this metric against the null hypothesis of 50% classification (using a single-sample *t*-test), in addition to computing Pearson correlations with scores on the BDI-II and GAF, age at onset of depression, and years since first depressive episode.

### Regional graph analysis

To assess abnormalities in network-level properties of individual nodes (i.e., brain regions), we conducted a regional graph analysis including the assessment of *degree centrality*, or the number of neighbors for each node, across groups. *Degree centrality* was selected based on its simple interpretation and widespread use (31). Permutation *t*-tests were used for each comparison, and FDR (*q* = 0.05) was used to control for false positive inflation as a result of multiple statistical tests [i.e., 68 tests, one per region; (36)].

## Results

### Demographics and clinical attributes

The depressed and non-depressed participants did not differ in level of education [χ^2^(3) = 1.83, *p* > 0.60], handedness [χ^2^(1) = 0.15, *p* > 0.70], or age [*t*(30) = −1.53, *p* > 0.10]. Not surprisingly, the MDD group obtained higher BDI-II and HAM-D scores, and lower GAF scores, than did the control group [BDI-II: *t*(30) = 16.59, *p* < 0.001; HAM-D: *t*(30) = 15.20, *p* < 0.001; GAF: *t*(30) = 14.36, *p* < 0.001; Table 2]. Based on standard BDI-II score cutoffs [moderate depression = 20–28; severe depression = 29–63; (17)], our depressed sample spans moderate to severe levels of depression (minimum score = 22; maximum score = 43). Three depressed participants were currently taking one or more psychotropic medications, including Venlafaxine and Sertraline, and three depressed participants were currently receiving psychotherapy. In addition, 7 of the 14 MDD participants met criteria for one or more anxiety disorders (Table 3).

**Table 2:** **Demographic information by group**.

	CTL (*N* = 18)				MDD (*N* = 14)				*p*-value
Sex: male/female	0		18		0		14		=1.00^§^
Age: years, *M*/*SD*/min/max	30.4	10.2	18.9	52.1	35.6	8.4	22.8	48.5	>0.10*
BDI-II: *M*/*SD*/min/max	2.2	3.2	0	11	31.7	6.6	22	43	<0.001*
HAM-D: *M*/*SD*/min/max	1.4	2.2	0	6	18.6	4.2	14	26	<0.001*
GAF: *M*/*SD*/min/max	87.8	7.0	75	99	53.0	6.6	35	60	<0.001*
Age of depression onset (years): *M*/*SD*/min/max	NA				16.3	6.8	3	26	NA
Years since depression onset: *M*/*SD*/min/max	NA				18.2	11.0	3	39	NA
Duration of current episode (months): *M*/*SD*/min/max	NA				10.2	12.3	2	47	NA
Handedness: left/right	2		16		1		13		>0.70^§^
Level of education^&^: *M*/*SD*/min/max	6.6	1.5	4	8	7.2	1.1	4	8	>0.60^§^

*CTL, control group; MDD, Major Depressive Disorder group; M, mean; SD, standard deviation; BDI-II, Beck Depression Inventory-II; HAM-D, Hamilton Rating Scale for Depression; GAF, Global Assessment of Functioning*.

*^&^Level of education was quantified as follows an individual having finished: elementary school received education score, 1, junior high school, 2, high school, 3, some college, 4, technical school, 5, junior college, 6, 4-year college, 7, graduate or professional education, 8*.

*Computed using two-sample *t*-tests.

*^§^Computed using chi-square test*.

**Table 3:** **MDD group psychiatric comorbidities**.

Psychiatric comorbidities	Number of MDD participants	% of MDD group
Any psychiatric comorbidities	7	50.0
Bulimia nervosa	1	7.1
General anxiety disorder	3	21.4
Panic disorder	2	14.3
Post-traumatic stress disorder	2	14.3
Social phobia	4	28.6
Specific phobia	2	14.3

*MDD, major depressive disorder group*.

### Global graph metrics

#### Univariate analyses of global graph metrics

Following previous structural graph analyses (11–13), we conducted univariate analyses on the global graph metrics examined in this study to assess the relations of specific global graph metrics to MDD. *Global flow coefficient* was the only metric that yielded an uncorrected permutation *t*-test *p*-value of <0.05; after FDR-correction, no permutation *t*-test comparing global graph metrics between groups reached significance (Table 4).

**Table 4:** **Univariate results comparing graph metrics between groups**.

Graph metric	CTL		MDD		*p*-value*
	*M*	*SD*	*M*	*SD*	
*Assortativity*	−0.062	0.022	−0.065	0.038	0.822
*Characteristic path length*	1.916	0.029	1.907	0.019	0.314
*Global betweenness*	62.921	2.649	62.559	1.492	0.659
*Global efficiency*	0.590	0.006	0.593	0.004	0.074
*Global flow coefficient*	0.329	0.013	0.339	0.013	0.039
*Global total flow*	1.441	4.592	1.441	3.962	0.991
*Modularity*	0.334	0.042	0.354	0.039	0.167
*Small-worldness*	1.548	0.039	1.575	0.040	0.067
*Transitivity*	0.548	0.012	0.543	0.548	0.187

*CTL, control group; MDD, major depressive disorder group; M, mean; SD, standard deviation*.

*p-values computed using permutation *t*-tests. No test reached significance after FDR multiple comparison correction.

#### General SVM classification performance

To assess the utility of SVM with global graph metric features for classifying depression, we assessed performance of the SVM set that included all nine metrics using the sign test. This test reached statistical significance (71.88% general accuracy, 71.43% sensitivity, 72.22% specificity; sign test: *p* < 0.025). In addition, we counted the number of sets of SVMs (using features from all combinations of the 9 metrics) that reached significance (i.e., 22 or more correct classifications from 32 folds during cross-validation). This count was then tested statistically using the binomial test with an expected outcome of 5% of tests reaching significance. A total of 228 of the 511 sets of SVMs reached significance (binomial test: *p* < 0.001).

#### SVM performance associated with specific graph metrics

To evaluate the utility of specific global graph metrics for depression classification, we first computed feature weights for graph metrics in the SVM set that included all nine graph metrics, and then averaged their ranks across folds. This analysis indicated that *small-worldness* had the highest average feature weight rank (indicating that it was the most important feature for classification), followed by *global efficiency* and *modularity* (Table 5). Second, we compared the SVM set accuracies associated with different global graph metrics (i.e., the accuracies of SVM sets that included the given metric; Table 5). The highest mean classification accuracy was associated with SVM sets that included *small-worldness*, followed by *global flow coefficient* (permutation *t*-test: *p* < 0.001).

**Table 5:** **Accuracy of SVMs sorted by individual graph metric and feature weight ranks for SVM set with all features**.

Graph metric	Across all SVM sets			SVM set with all features		
	Accuracy		SVM count*	Rank across folds^a^		Ranked means^b^
	*M*%	*SD*%		*M*	*SD*	
*Assortativity*	64.40	9.3	118	5.78	1.21	7
*Characteristic path length*	65.39	8.3	123	5.13	1.34	4
*Global betweenness*	64.29	8.8	115	8.47	0.57	9
*Global efficiency*	65.75	7.9	122	2.13	0.42	2
*Global flow coefficient*	66.28	6.9	132	5.53	1.46	6
*Global total flow*	66.25	8.7	134	8.34	0.75	8
*Modularity*	65.76	8.2	122	3.22	0.79	3
*Small-worldness*	68.88	6.3	169	1.03	0.18	1
*Transitivity*	65.75	8.8	135	5.38	1.31	5

*SVM count, number of significant classifications (i.e., the number of sets including the given graph metric that reached significance as defined by the sign test) out of a total of 256 per metric*.

*All *p*-values <0.001 (as assessed using the binomial test).

*^a^Mean (M) and standard deviation (SD) of feature weight ranks across folds, for the SVM set with all nine graph metric features*.

*^b^Ranked mean ranked feature weights across folds for the SVM set with all nine graph metric features*.

#### Additional analyses of small-worldness

Given that *small-worldness* performed best and had the highest ranked feature weight, we conducted further analyses to better understand the relation of this metric to the classification of depression. Using a single-sample *t*-test, we compared the accuracies associated with *small-worldness* to the null hypothesis of a classification accuracy of 50% (i.e., chance in a binary decision). This test indicated that the classification accuracies were significantly greater than chance, *t*(255) = 9.1, *p* < 0.001. Neither the SVM set that included only *small-worldness* (59.4% accuracy; sign test: *p* > 0.35) nor comparing *small*-*worldness* between groups reached significance (Table 4). In addition, we conducted correlations between *small-worldness* and clinical variables within the MDD group. None of these tests yielded statistically significant results: BDI-II, *r* = 0.11, *p* > 0.70; GAF, *r* = 0.23, *p* > 0.40; age of onset of depression, *r* = 0.41, *p* > 0.10; years since first episode, *r* = 0.04, *p* > 0.85.

### Regional graph metrics

**Figure 1:**
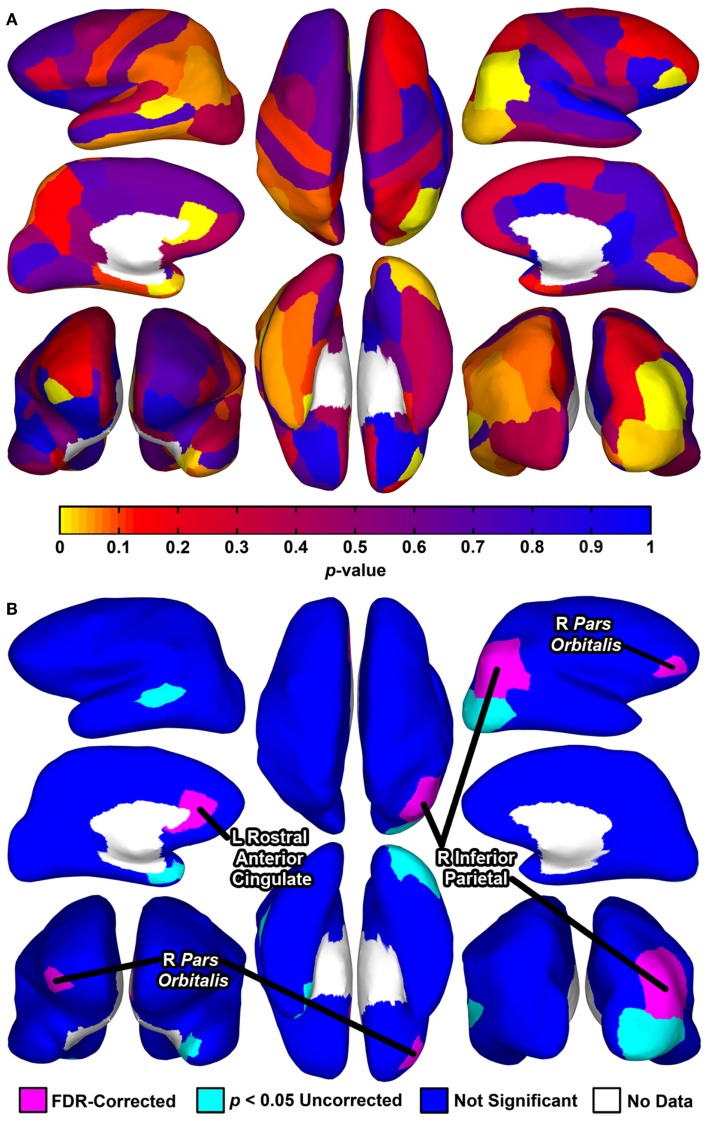
**Cortical surface renderings of regional graph analysis of *degree centrality* between groups**. Data are rendered on the cortical surface of an example participant. **(A)** *p*-values assessed using permutation *t-*tests between groups projected to the cortical surface for each of the 68 regions. **(B)** Statistical significance of the tests depicted in **(A)**. Seven regions exhibited *p*-values <0.05 uncorrected, with three of these regions significant after correction for multiple comparisons using a FDR (false discovery rate) procedure (for means and SD, see Table 6). For **(A,B)**, upper left, left hemisphere lateral view; middle left, left hemisphere medial view; bottom left, bilateral anterior view (right hemisphere on left side); upper middle, bilateral superior view (left hemisphere on left side); bottom middle, bilateral inferior view (left hemisphere on left side); upper right, right hemisphere lateral view; middle right, right hemisphere medial view; bottom right, bilateral posterior view.

Permutation *t*-tests yielded uncorrected *p*-values of <0.05 for group comparisons of *degree centrality* for seven brain regions (Table 6; Figure 1). Three of these tests reached significance after correction for multiple comparisons using FDR: the right *pars orbitalis* of the right ventrolateral prefrontal cortex (VLPFC), right inferior parietal cortex, and left rostral anterior cingulate.

**Table 6:** **Regional *degree centrality* by group**.

Region	CTL		MDD		*p*-value*
	*M*	*SD*	*M*	*SD*	
Left banks superior temporal sulcus	15.6	2.3	13.1	3.4	0.014
Left entorhinal	3.7	2.9	7.3	3.5	0.003
Left rostral anterior cingulate	20.5	1.2	22.1	1.3	0.002**
Left temporal pole	5.8	2.1	7.4	1.6	0.024
Right inferior parietal	21.4	1.9	18.2	1.7	<0.001**
Right lateral occipital	17.0	1.7	15.7	1.0	0.021
Right *Pars orbitalis*	5.5	0.7	6.6	1.1	<0.001**

*M, mean; SD, standard deviation*.

*p-value as computed using permutation two-sample, two-tailed *t*-tests.

**Significant *p*-values after correction for multiple comparisons using a false discovery rate (FDR) procedure (*q* = 0.05).

## Discussion

Despite considerable interest in understanding network-level brain abnormalities in MDD, investigators have not identified tractography-based whole-brain graph metrics that differentiate individuals diagnosed with this disorder from healthy controls. In the present study, we show, first, that individuals diagnosed with MDD can be differentiated from healthy controls using a collection of whole-brain graph metrics derived from the diffusion-tractography-based connectomes that can be optimally combined using the results from SVMs. Second, using feature scoring techniques, we found that *small-worldness* was associated with the highest classification accuracies and largest feature weights. Finally, our results indicate that regional connectedness is abnormal in MDD. That is, we used a local graph analysis approach of *degree centrality* to compare regional connectedness in MDD to healthy controls and identified three brain regions that differentiated the MDD group from the healthy controls: right *pars orbitalis*, right inferior parietal cortex, and left rostral anterior cingulate. Whereas the parietal region exhibited reduced connectivity in MDD, the *pars orbitalis* and rostral anterior cingulate exhibited greater connectivity.

Three previous studies that have used tractography-based graph analyses in MDD found that whole-brain graph metrics did not differentiate depressed participants from healthy controls (11–13). Indeed, our univariate analyses also did not yield whole-brain abnormalities associated with MDD. Therefore, our findings build on previous results by suggesting that *combinations* of tractography-based graph metrics are critical for classification of this disorder, and that multivariate machine learning techniques can be used to identify these patterns.

Whole-brain graph metrics derived from other imaging methods (e.g., rs-fMRI or inter-regional volume correlations) have also been found to identify abnormalities in MDD. For example, using rs-fMRI, Meng et al. (42) found that MDD was associated with reduced *global efficiency* and increased *global betweenness* and *path length*; similarly, Singh et al. (43) used inter-regional volume correlations and found that *global clustering coefficient* was reduced in MDD. Notably, several studies have reported using machine learning to classify depressed vs. healthy individuals using graph metrics derived from rs-fMRI (44, 45). Graph metrics derived from different neuroimaging and electrophysiology modalities may provide unique and potentially complementary information about abnormal brain networks in depression. Future research may benefit from explicitly comparing graph metrics derived from different modalities.

The current findings support the possibility of combining graph metrics with machine learning to identify biosignatures for use in a clinical context, for purposes of prevention, diagnosis, and treatment. For such a use to be viable, it will be necessary to improve classification accuracies for MDD. Given that the methods used to define nodes have been shown to affect global graph metrics (46), improved classification may be achieved by determining the most effective technique for node identification. For example, it is not clear which of the Desikan–Killiany and Destrieux cortical parcellations are better for node identification for the purposes of classification (23, 47), or, more generally, whether functionally or anatomically defined nodes might yield stronger classification performance. Classification may also be improved by using features derived from multi-modal data and by utilizing more sophisticated machine learning methods. For example, feature selection techniques may improve performance by removing redundant features and noise, and some classification methods may be more useful than others [e.g., SVMs, relevance vector machines (RVMs), Gaussian process classifiers (GPCs); (48)].

Based on feature scoring, *small-worldness* was associated with the highest classification accuracies and largest feature weights. Given that *small-worldness* was computed as the balance between a metric of segregation (*transitivity*) and integration (*characteristic path length*), it may offer more information than either segregation or integration (49). In fact, as additional support for this formulation, we found a statistical trend in our univariate analysis of *small-worldness* (*p* < 0.10), but not of *transitivity* or *characteristic path length*. Despite a potential relation between *small-worldness* and MDD, in the current study we did not find statistically significant relations between *small-worldness* and BDI-II scores, age of depression onset, or years since first depressive episode. This may be a result of low statistical power. Thus, future research might profitably examine the relation between *small-worldness* and symptoms and characteristics of MDD.

Our analysis of *degree centrality* revealed abnormal connectivity of the right *pars orbitalis*, right inferior parietal cortex, and left rostral anterior cingulate. The FreeSurfer rostral anterior cingulate cortex prominently includes sgACC, one of the most consistently implicated regions in MDD. For example, neuroimaging studies have found that depressed individuals tend to exhibit increased sgACC activity, and that the extent of this abnormality may be reduced with pharmacological treatment (50). Moreover, the *pars orbitalis*, Brodmann’s area (BA) 47 or anterior [VLPFC; (51)], is included in orbitofrontal cortex (OFC), which is posited to be involved in emotion processing and has abnormal volume and functional connectivity in MDD (52–55). Finally, with respect to the right inferior parietal region, the parietal cortex has shown MDD-related abnormalities in cognitive and affective tasks (56, 57). Specifically, the right parietal cortex may be implicated in impaired emotion processing and decreased arousal (58, 59). Our regional graph analyses expand these findings to a tractography-based network context, further supporting the importance of these regions in depression. Because *degree centrality* indexes how communication of a given area with the rest of the brain may be facilitated or reduced, future studies should relate these MDD-related abnormalities (i.e., facilitated communication of the right *pars orbitalis* and left rostral anterior cingulate, and reduced communication of right inferior parietal cortex) to specific cognitive and affective processes associated this disorder. For example, future studies could relate increased sgACC network connectivity in MDD with abnormal experience of emotion (60).

One study has documented that individuals with remitted geriatric depression exhibit reduced network strength and *global efficiency*, and increased *characteristic path length* (61). Thus, future research might assess the relation of global network properties to remission and age across the lifespan (i.e., in childhood, adolescence, adulthood, and old age). Given the heterogeneity of MDD, and the recent proposal by the NIMH supporting the use of Research Domain Criteria (RDoC), it will also be important in future research to relate graph metrics to clinical signs and symptoms, and to behavior and brain processes in a transdiagnostic, spectrum-based manner, and to use multiple units of analysis. This approach promises to increase our understanding of basic network-level abnormalities and their relation to psychopathology.

We should note four limitations of this study: (1) half of the MDD participants in this study were diagnosed with comorbid anxiety disorders; (2) three MDD participants were taking psychotropic medications; (3) all participants were female; and, (4) our sample size is relatively small (*N* = 32). Thus, it will be important in future to examine the potential influence of anxiety comorbidities, pharmacological agents, age, and gender on measures of network connectivity in depression, in addition to replicating our current findings in a larger cohort of depressed and healthy individuals.

## Conclusion

The present study is important in describing the first use of global tractography-based graph metrics for the classification of depression, and the identification of *small-worldness* as the most useful graph metric for this purpose. We further identified the right *pars orbitalis*, right inferior parietal cortex, and left rostral anterior cingulate as exhibiting abnormal connectivity in MDD. These findings highlight important directions for future research, including the assessment of graph metrics across different imaging modalities, optimizing classification (e.g., atlas selection), relations of graph metrics to clinical signs and symptoms, psychiatric comorbidities, and psychotropic agents.

## Conflict of Interest Statement

The authors declare that the research was conducted in the absence of any commercial or financial relationships that could be construed as a potential conflict of interest.

## Footnotes

^1^http://fsl.fmrib.ox.ac.uk/fsl/fslwiki/

^2^http://surfer.nmr.mgh.harvard.edu/

## References

[1] World Health Organization. World Health Organization Depression Fact sheet No 369. (2012). Available from: http://www.who.int/mediacentre/factsheets/fs369/en/

[2] Whiteford HA Degenhardt L Rehm J Baxter A Ferrari AJ Erskine HE Global burden of disease attributable to mental and substance use disorders: findings from the Global Burden of Disease Study 2010. J Lancet (2013) 382:1575–86. 10.1016/S0140-6736(13)61611-6 23993280

[3] Sheline YI Barch DM Donnelly JM Ollinger JM Snyder AZ Mintun MA. Increased amygdala response to masked emotional faces in depressed subjects resolves with antidepressant treatment: an fMRI study. Biol Psychiatry (2001) 50:651–8. 10.1016/S0006-3223(01)01263-X 11704071

[4] Siegle GJ Steinhauer SR Thase ME Stenger VA Carter CS. Can’t shake that feeling: event-related fMRI assessment of sustained amygdala activity in response to emotional information in depressed individuals. Biol Psychiatry (2002) 51:693–707. 10.1016/S0006-3223(02)01314-8 11983183

[5] Wang L Hermens DF Hickie IB Lagopoulos J A systematic review of resting-state functional-MRI studies in major depression. J Affect Disord (2012) 142:6–12. 22858266 10.1016/j.jad.2012.04.013

[6] Sexton CE Mackay CE Ebmeier KP A systematic review of diffusion tensor imaging studies in affective disorders. Biol Psychiatry (2009) 66:814–23 10.1016/j.biopsych.2009.05.024 19615671

[7] Murphy ML Frodl T. Meta-analysis of diffusion tensor imaging studies shows altered fractional anisotropy occurring in distinct brain areas in association with depression. Biol Mood Anxiety Disord (2011) 1:3. 10.1186/2045-5380-1-3 22738088 PMC3377129

[8] Liao Y Huang X Wu Q Yang C Kuang W Du M Is depression a disconnection syndrome? Meta-analysis of diffusion tensor imaging studies in patients with MDD. J Psychiatry Neurosci (2013) 38:49–56 10.1503/jpn.110180 22691300 PMC3529219

[9] van den Heuvel MP Sporns O Network hubs in the human brain. Trends Cogn Sci (2013) 17:683–96 10.1016/j.tics.2013.09.012 24231140

[10] Sporns O. Network attributes for segregation and integration in the human brain. Curr Opin Neurobiol (2013) 23:162–71. 10.1016/j.conb.2012.11.015 23294553

[11] Korgaonkar MS Fornito A Williams LM Grieve SM. Abnormal structural networks characterize major depressive disorder: a connectome analysis. Biol Psychiatry (2014) 76:567–74. 10.1016/j.biopsych.2014.02.018 24690111

[12] Qin J Wei M Liu H Yan R Luo G Yao Z Abnormal brain anatomical topological organization of the cognitive-emotional and the frontoparietal circuitry in major depressive disorder. Magn Reson Med (2013) 72:1397–407. 10.1002/mrm.25036 24273210

[13] Ajilore O Lamar M Leow A Zhang A Yang S Kumar A Association of brain network Ef. Am J Geriatr Psychiatry (2014) 22:102–10 10.1016/j.jagp.2013.10.004 24200596 PMC3947188

[14] Cortes C Vapnik V Support-vector networks. Mach Learn (1995) 20:273–97 10.1023/A:1022627411411

[15] First MB Dibbon M Spitzer RL Williams JB Structured clinical interview for DSM-IV-TR. Washington, DC: American Psychiatric Association (2004).

[16] Hamilton M A rating scale for depression. J Neurol Neurosurg Psychiatry (1960) 23:56–62 10.1136/jnnp.23.1.56 14399272 PMC495331

[17] Beck AT Steer RA Brown GK Manual for the Beck Depression Inventory-II. San Antonio, TX: Psychological Corporation (1996).

[18] Endicott J Spitzer RL Fleiss JL Cohen J. The global assessment scale: a procedure for measuring overall severity of psychiatric disturbance. Arch Gen Psychiatry (1976) 33:766–71. 10.1001/archpsyc.1976.01770060086012 938196

[19] Friston KJ Ashburner J Generative and recognition models for neuroanatomy. Neuroimage (2004) 23:21–4 10.1016/j.neuroimage.2004.04.021 15325348

[20] Basser PJ Pierpaoli C. Microstructural and physiological features of tissues elucidated by quantitative-diffusion-tensor MRI. J Magn Reson B (1996) 111:209–19. 10.1006/jmrb.1996.0086 8661285

[21] Aganj I Lenglet C Jahanshad N Yacoub E Harel N Thompson PM A Hough transform global probabilistic approach to multiple-subject diffusion MRI tractography. Med Image Anal (2011) 15:414–25. 10.1016/j.media.2011.01.003 21376655 PMC3115463

[22] Prasad G Nir TM Toga AW Thompson PM. Tractography density and network measures in Alzheimer’s disease. Proc IEEE Int Symp Biomed Imaging (2013) 2013:692–5. 25404994 10.1109/ISBI.2013.6556569 PMC4232938

[23] Desikan RS Ségonne F Fischl B Quinn BT. An automated labeling system for subdividing the human cerebral cortex on MRI scans into gyral based regions of interest. Neuroimage (2006) 31:968–80. 10.1016/j.neuroimage.2006.01.021 16530430

[24] Han X Jovicich J Salat D van der Kouwe A Quinn B Czanner S Reliability of MRI-derived measurements of human cerebral cortical thickness: the effects of field strength, scanner upgrade and manufacturer. Neuroimage (2006) 32:180–94. 10.1016/j.neuroimage.2006.02.051 16651008

[25] Jovicich J Marizzoni M Sala-Llonch R Bosch B Bartrés-Faz D Arnold J Brain morphometry reproducibility in multi-center 3T MRI studies: a comparison of cross-sectional and longitudinal segmentations. Neuroimage (2013) 83:472–84. 10.1016/j.neuroimage.2013.05.007 23668971

[26] Ardekani BA Guckemus S Bachman A Hoptman MJ Wojtaszek M Nierenberg J. Quantitative comparison of algorithms for inter-subject registration of 3D volumetric brain MRI scans. J Neurosci Methods (2005) 142:67–76. 10.1016/j.jneumeth.2004.07.014 15652618

[27] Klein A Andersson J Ardekani BA Ashburner J Avants B Chiang M-C Evaluation of 14 nonlinear deformation algorithms applied to human brain MRI registration. Neuroimage (2009) 46:786–802. 10.1016/j.neuroimage.2008.12.037 19195496 PMC2747506

[28] Sporns O Networks of the Brain. Cambridge, MA: MIT Press (2011)

[29] Dennis EL Jahanshad N Toga AW McMahon KL de Zubicaray GI Martin NG Test-retest reliability of graph theory measures of structural brain connectivity. In: Ayache N Delingette H Golland P Mori K, editors. Medical Image Computing and Computer-Assisted Intervention – MICCAI 2012 Lecture Notes in Computer Science. Berlin: Springer (2012). p. 305–12. 10.1007/978-3-642-33454-2_38 PMC4039303 23286144

[30] Monti MM Lutkenhoff ES Rubinov M Boveroux P Vanhaudenhuyse A Gosseries O Dynamic change of global and local information processing in propofol-induced loss and recovery of consciousness. PLoS Comput Biol (2014) 9:e1003271. 10.1371/journal.pcbi.1003271 24146606 PMC3798283

[31] Rubinov M Sporns O. Complex network measures of brain connectivity: uses and interpretations. Neuroimage (2010) 52:1059–69. 10.1016/j.neuroimage.2009.10.003 19819337

[32] Honey CJ Kötter R Breakspear M Sporns O. Network structure of cerebral cortex shapes functional connectivity on multiple time scales. Proc Natl Acad Sci U S A (2007) 104:10240–5. 10.1073/pnas.0701519104 17548818 PMC1891224

[33] Lord L-D Allen P Expert P Howes O Lambiotte R McGuire P Characterization of the anterior cingulate’s role in the at-risk mental state using graph theory. Neuroimage (2011) 56:1531–9. 10.1016/j.neuroimage.2011.02.012 21316462

[34] Newman M The structure and function of complex networks. SIAM Rev Soc Ind Appl Math (2003) 45:167–256 10.1137/S003614450342480

[35] Humphries MD Gurney K. Network “small-world-ness”: a quantitative method for determining canonical network equivalence. PLoS One (2008) 3:e0002051. 10.1371/journal.pone.0002051 18446219 PMC2323569

[36] Benjamini Y Hochberg Y Controlling the false discovery rate: a practical and powerful approach to multiple testing. J R Stat Soc Ser A (1995) 57:289–300.

[37] Redlich R Almeida JJR Grotegerd D Opel N Kugel H Heindel W Brain morphometric biomarkers distinguishing unipolar and bipolar depression: a voxel-based morphometry–pattern classification approach. JAMA Psychiatry (2014) 71:1222–30. 10.1001/jamapsychiatry.2014.1100 25188810 PMC5538312

[38] Sun D van Erp TGM Thompson PM Bearden CE Daley M Kushan L Elucidating a magnetic resonance imaging-based neuroanatomic biomarker for psychosis: classification analysis using probabilistic brain atlas and machine learning algorithms. Biol Psychiatry (2009) 66:1055–60. 10.1016/j.biopsych.2009.07.019 19729150 PMC3192809

[39] Orrù G Pettersson-Yeo W Marquand AF Sartori G Mechelli A. Neuroscience and biobehavioral reviews. Neurosci Biobehav Rev (2012) 36:1140–52. 10.1016/j.neubiorev.2012.01.004 22305994

[40] Hastie T Tibshirani R Friedman J The Elements of Statistical Learning. (Vol. 2). New York, NY: Springer (2009).

[41] De Martino F Valente G Staeren N Ashburner J Goebel R Formisano E. Combining multivariate voxel selection and support vector machines for mapping and classification of fMRI spatial patterns. Neuroimage (2008) 43:44–58. 10.1016/j.neuroimage.2008.06.037 18672070

[42] Meng C Brandl F Tahmasian M Shao J Manoliu A Scherr M Aberrant topology of striatum’s connectivity is associated with the number of episodes in depression. Brain (2014) 137:598–609. 10.1093/brain/awt290 24163276

[43] Singh MK Kesler SR Hosseini SMH Kelley RG Amatya D Hamilton JP Anomalous gray matter structural networks in major depressive disorder. Biol Psychiatry (2013) 74:777–85. 10.1016/j.biopsych.2013.03.005 23601854 PMC3805751

[44] Lord A Horn D Breakspear M Walter M. Changes in community structure of resting state functional connectivity in unipolar depression. PLoS One (2012) 7:e41282. 10.1371/journal.pone.0041282 22916105 PMC3423402

[45] Zeng LL Shen H Liu L Wang L Li B Fang P Identifying major depression using whole-brain functional connectivity: a multivariate pattern analysis. Brain (2012) 135:1498–507. 10.1093/brain/aws059 22418737

[46] Zalesky A Fornito A Harding IH Cocchi L Yucel M Yücel M Whole-brain anatomical networks: does the choice of nodes matter? Neuroimage (2010) 50:970–83. 10.1016/j.neuroimage.2009.12.027 20035887

[47] Destrieux C Fischl B Dale A Halgren E. Automatic parcellation of human cortical gyri and sulci using standard anatomical nomenclature. Neuroimage (2010) 53:1–15. 10.1016/j.neuroimage.2010.06.010 20547229 PMC2937159

[48] Mwangi B Tian TS Soares JC A review of feature reduction techniques in neuroimaging. Neuroinformatics (2013) 12:229–44 10.1007/s12021-013-9204-3 24013948 PMC4040248

[49] Watts DJ Strogatz SH. Collective dynamics of |[lsquo]|small-world|[rsquo]| networks. Nature (1998) 393:440–2. 10.1038/30918 9623998

[50] Hamani C Mayberg H Stone S Laxton A Haber S Lozano AM. The subcallosal cingulate gyrus in the context of major depression. Biol Psychiatry (2011) 69:301–8. 10.1016/j.biopsych.2010.09.034 21145043

[51] Levy BJ Wagner AD. Cognitive control and right ventrolateral prefrontal cortex: reflexive reorienting, motor inhibition, and action updating. Ann N Y Acad Sci (2011) 1224:40–62. 10.1111/j.1749-6632.2011.05958.x 21486295 PMC3079823

[52] Frodl T Bokde ALW Scheuerecker J Lisiecka D Schoepf V Hampel H Functional connectivity bias of the orbitofrontal cortex in drug-free patients with major depression. Biol Psychiatry (2010) 67:161–7. 10.1016/j.biopsych.2009.08.022 19811772

[53] Lacerda ALT Keshavan MS Hardan AY Yorbik O Brambilla P Sassi RB Anatomic evaluation of the orbitofrontal cortex in major depressive disorder. Biol Psychiatry (2004) 55:353–8. 10.1016/j.biopsych.2003.08.021 14960287

[54] Ballmaier M Toga AW Blanton RE. Anterior cingulate, gyrus rectus, and orbitofrontal abnormalities in elderly depressed patients: an MRI-based parcellation of the prefrontal cortex. Am J Psychiatry (2004) 161:99–108. 10.1176/appi.ajp.161.1.99 14702257

[55] Bremner JD Vythilingam M Vermetten E Nazeer A Reduced volume of orbitofrontal cortex in major depression. Biol Psychiatry (2002) 51:273–9 10.1016/S0006-3223(01)01336-1 11958777

[56] Liotti M Mayberg HS. The role of functional neuroimaging in the neuropsychology of depression. J Clin Exp Neuropsychol (2001) 23:121–36. 10.1076/jcen.23.1.121.1223 11320448

[57] Mayberg HS. Limbic-cortical dysregulation: a proposed model of depression. J Neuropsychiatr (1997) 9:471–81. 10.1176/jnp.9.3.471 9276848

[58] Heller W Neuropsychological mechanisms of individual differences in emotion, personality, and arousal. Neuropsychology (1993) 7:476–89 10.1037/0894-4105.7.4.476

[59] Stewart JL Towers DN Coan JA Allen JJB. The oft-neglected role of parietal EEG asymmetry and risk for major depressive disorder. Psychophysiology (2010) 48:82–95. 10.1111/j.1469-8986.2010.01035.x 20525011 PMC3000438

[60] Price JL Drevets WC Neurocircuitry of mood disorders. Neuropsychopharmacology (2009) 35:192–216 10.1038/npp.2009.104 19693001 PMC3055427

[61] Bai F Shu N Yuan Y Shi Y Yu H Wu D Topologically convergent and divergent structural connectivity patterns between patients with remitted geriatric depression and amnestic mild cognitive impairment. J Neurosci (2012) 32:4307–18. 10.1523/JNEUROSCI.5061-11.2012 22442092 PMC6621223

